# Rethinking the COVID-19 Pandemic: Back to Public Health

**DOI:** 10.5334/aogh.3084

**Published:** 2020-10-08

**Authors:** Téa Collins, Svetlana Akselrod, Ashley Bloomfield, Amiran Gamkrelidze, Zsuzsanna Jakab, Erika Placella

**Affiliations:** 1 World Health Organization, CH; 2 Ministry of Health, NZ; 3 National Center for Disease Control and Public Health, GE; 4 Swiss Agency for Development and Cooperation, CH

## Abstract

The COVID-19 pandemic has highlighted vast differences across countries in their responses to the emergency and their capacities to implement public health measures that could slow the progression of the disease.

As public health systems are the first line of defense during pandemics, it has become clear that sustained investment in strengthening public health infrastructure is a major need in all countries, irrespective of income levels. Drawing on the successful experiences of Switzerland, Georgia, and New Zealand in dealing with COVID-19, we suggest prioritizing core public health capacities with links to the International Health Regulations, improving international cooperation, coordination, and multisectoral action, addressing health inequities by targeting vulnerable groups, and enhancing health literacy, including through sophisticated and sustained communication campaigns to build resilience. These measures will ensure that health systems and communities will be better prepared for the disruptions that future disease outbreaks will inevitably bring.

## Introduction

Over the past several months, the world has been living in a state of public health emergency due to the COVID-19 pandemic. Since the beginning of the year, the Severe Acute Respiratory Syndrome Coronavirus 2 (SARS-CoV-2) has been spreading rapidly, infecting, at the time of this writing, nearly 34 million people and taking more than a million lives in 216 countries and territories, threatening global health security, stretching national healthcare systems beyond their capacities and impeding countries’ economic growth and prosperity [1].

While some countries and territories have responded well to COVID-19, many of the shortcomings of the current pandemic response could have been mitigated significantly had public health systems been better prepared. Rapid advances in medicine, highly-specialized treatments, and technological innovation over the past decades have increased demand for fast fixes to health problems and tended to sideline public health responses, resulting in chronic underinvestment in core public health capacities, including pandemic preparedness and response, even in many wealthy nations [2].

Experiences from this and previous outbreaks (e.g., SARS, Ebola, Zika, Influenza) have taught us that viruses do not respect borders, that both rich and poor countries are vulnerable, that disadvantaged populations are hardest hit, and that weak public health capacities for disease surveillance, monitoring and reporting are a risk to health security. Global solidarity and cooperation among governments, civil society, and the private sector are essential to overcome the health challenges of the 21st century [3].

The purpose of this paper is to highlight the overarching areas that we believe need to be prioritized to enhance governments’ ability for effective prevention, alert, and response to emergencies through public health approaches, and to improve the baseline health and well-being of their populations, so they become more resilient to health shocks that disease outbreaks bring.

We suggest four main areas of focus: (i) strengthening core public health capacities and increasing investments in public health emergency preparedness; (ii) enhancing international cooperation and solidarity, multisectoral action and implementation of global commitments at the national level; (iii) reducing health inequities by prioritizing the needs of vulnerable populations; and (iv) improving health literacy and the responsiveness of the health system to people’s cultural and socioeconomic context. To support our arguments, we also provide the positive experiences of three countries – Switzerland, Georgia, and New Zealand – in their responses to the pandemic. We believe these examples offer useful lessons about the value of strong public health approaches and government commitment.

## 1. Public Health Systems are Critical to Promote Health and Prevent Disease Outbreaks

### Public Health and Medicine – Two Sides of the Same Coin

Public health is a multidisciplinary field concerned with the understanding of the etiology and distribution of diseases in populations rather than individuals. It looks closely at environmental, social, economic, nutritional, and behavioral factors, applies systems thinking, and employs a range of social and community interventions to preserve and protect the health of whole communities. Even though there are differences across countries in how disease prevention, health promotion, emergency preparedness, social participation, and communication within public health are framed, all share the common elements of essential public health functions, including surveillance, governance and financing, health promotion, health protection/legislation, research and human resources [4]. Evidence suggests that essential public health functions offer the most cost-effective and sustainable way to achieve universal health coverage and the Sustainable Development Goals [5]. Clinical medicine, on the other hand, is mainly concerned with the diagnosis and treatment of individuals who are already suffering from ill-health [6].

Pandemic preparedness and response require close coordination between public health and clinical medicine in all stages of the pandemic, including strengthening the resilience of primary healthcare systems to respond to infectious disease outbreaks and ensure a functional referral to other levels of care [7] (Table 1).

**Table 1:** Coordinated Public Health and Medical Interventions for Emergency Preparedness and Response by Period.

*Pre-pandemic Period (before a Pandemic starts)*

Assessment of existing surveillance and recommended improvements for pre-pandemic and pandemic surveillance Assessment of community mitigation strategies and recommended improvements Stockpile building (antivirals, antibiotics, vaccines) Service continuity planning/hospital preparedness plans Public health workforce training Simulation exercises Risk transfer mechanism set-up Situational awareness
***Spark Period (when a Pandemic starts)***

Initial outbreak detection Pathogen characterization or laboratory confirmation Risk communication and community engagement Animal disease control Contact tracing, quarantine, and isolation Situational awareness
***Spread Period (after a Pandemic starts)***

Global pandemic declaration Risk communication & public information Contact tracing, quarantine, and isolation Physical distancing Stockpile deployment Vaccine or antiviral administration Care and treatment Situational awareness*

* Situational awareness includes passive and active animal and human disease surveillance and monitoring of public health facilities and resources. Source: Adapted from Madhav N, Oppenheim B, Gallivan M et al. Pandemics: Risks, Impacts, and Mitigation. In: Jamison DT, Gelband H, Horton S, Jha P, Laxminarayan R, Mock CN, and Nugent R, editors. Disease Control Priorities: Improving Health and Reducing Poverty. Disease Control Priorities (third edition). Volume 9. 2018; Washington, DC: World Bank. doi: 10.1596/978-1-4648-0527-1.

### Need for Greater Investment in Public Health Systems

Historically, the international community has vastly underestimated the risk that new and reemerging pathogens can pose globally. Even if a pandemic does not involve high mortality, the economic and social costs may still be significant. For example, the 2003 SARS pandemic, which encompassed four continents and 37 countries, resulted in a GDP loss of about US$4 billion in Hong Kong and China, US$5 billion in Singapore, and up to US$6 billion in Canada [8]. The Zika virus outbreak, affecting 76 countries, resulted in a loss of between US$7 billion and US$18 billion in Latin America and the Caribbean [9].

Globally, estimated losses from pandemics stand at US$60 billion direct costs per year, amounting to a projected US$6 trillion loss by the end of the 21st century. If indirect costs are counted, the annual number may go as high as US$490 billion [10]. Preliminary estimates indicate that the COVID-19 pandemic has already far exceeded these predictions and could itself cost up to US$10 trillion [11]. In contrast, a relatively modest investment of US$4.5 billion in public health systems, or about 65 cents per person globally, can strengthen global preparedness considerably and save many lives [12].

A sustainable commitment to prevention, including sufficient financing of public health infrastructure, may be politically challenging even in high-income countries. Public health is often considered a politically ‘soft target’ for budget cuts, as the benefits of population-level public health expenditure are long-term and often seem uncertain to policy-makers [13]. It is estimated that OECD countries allocate only 2% to 4% of total health expenditure to health promotion and disease prevention activities [14]. This relative underinvestment in public health compared with clinical services may not be wise, since investing in public health interventions has been shown to give fourfold economic returns to the health sector, and the wider economy for every dollar invested [15]. On average, individual-level interventions cost five times more than population-based measures [2]. Therefore, strengthening public health functions and reserving medical systems (especially hospital care) as a last resort for individual patients who need specialized care is justified from epidemiological as well as economic perspectives.

## 2. International Cooperation, Solidarity, and Multisectoral Action for Public Health

The World Health Organization (WHO) relies in large part on International Health Regulations 2005 (IHR), a legally binding framework agreed upon by 196 “State Parties,” to prevent and control the spread of diseases globally. In response to the IHR, many countries have developed pandemic preparedness plans and provide self-assessment annual reports to WHO on IHR implementation encompassing the core public health capacities across 13 dimensions, including *legislation and financing; coordination and national IHR focal point functions; zoonotic events, and the human-animal interface; food safety; laboratory capacity; surveillance; human resources; national health emergency framework; health service provision; risk communication; points of entry; chemical events, and radiation emergencies* [16]. The IHR core capacities are an integral part of essential public health operations, and their fulfillment is important for responding to public health emergencies as well as for strengthening overall national health systems and international cooperation.

However, the implementation of the IHR commitments has been slow. By the end of 2015, just 35% of countries had core capacities in place [17]. A review of national pandemic preparedness plans of 35 countries in the WHO African region showed that while most countries in the region had a plan (74%), the majority of these plans were inadequate and had not been updated over time. Also, all the plans were heavily dependent on external funding, with no sustainable budget for their implementation [18].

External assistance in resource-constrained countries is often critical to complement limited domestic public health resources, and strategies should be tailored to local needs and priorities [19]. The IHR currently mandates that technical support for surveillance, epidemiology, and other core capacities be provided by high-income countries to low-income countries. During a pandemic, mobilizing bilateral funds may be difficult when all countries are busy taking care of their own needs. However, the recent pledge by the G20 member states of USD21 billion to fight the COVID-19 pandemic demonstrated the spirit of solidarity and collective action needed to address global health emergencies [20].

Switzerland has also adopted a comprehensive international cooperation strategy that has placed the response to COVID-19 centerstage (**Box 1**).

**Box 1: Stepping up to the plate: Switzerland prioritizes international solidarity to strengthen the resilience of health systems to health shocks in countries with fragile economies.**

Switzerland’s *International Cooperation Strategy 2021–2024* emphasizes equitable access to quality basic services, particularly in the area of health. *Switzerland’s Foreign Health Policy 2019–2024* also aims to strengthen the resilience to health shocks of health systems in low- and middle-income countries. Finally, the prevention of epidemics in these countries, as well as in countries weakened by conflicts and other chronic crises, is one of the priorities of the Swiss Agency for Development Cooperation *(SDC) Health Policy*.

Thanks to Switzerland’s strategic role and positioning in global health, its presence in the health sector in various countries and the flexibility of its instruments, Switzerland was able to quickly contribute to the fight against the pandemic. The Federal Council decided to respond to various appeals from international organizations and forums (UN, ICRC, G20, etc.) with CHF 400 million, distributed as follows:

– An interest-free loan of CHF 200 million to the International Committee of the Red Cross (ICRC).
– A contribution of CHF 25 million to the International Monetary Fund (IMF) Disaster Relief and Response Trust Fund.
– Other contributions to the ICRC, the International Federation of Red Cross and Red Crescent Societies, and the Access to COVID-19 Tools Accelerator initiative (diagnostics, therapies and vaccines), as well as contributions of bilateral development cooperation and humanitarian aid totaling CHF 175 million.
– In addition, various ongoing programmes financed from the existing budget have been adapted to integrate a crisis response. In Burkina Faso and Chad, for example, kits for the local production of disinfection solutions have been provided to various health facilities. In Kosovo, awareness-raising messages were developed and disseminated to the population, targeting the most vulnerable communities.
– The SDC is contributing to the strengthening of systems for the prevention, surveillance, control and response to COVID-19 in Laos and the Horn of Africa region, in particular through the provision of reliable screening tests. In Tanzania and Eastern DRC, the SDC supports the improvement of local capacities for social mobilization of communities and communication of the risks linked to the virus. This includes raising awareness on physical distancing and handwashing, broadcasting radio messages in local languages and distributing information brochures with pictograms to make it easier to understand and comply with the recommendations.
– Swiss-based non-governmental organizations supported by the SDC are also adapting their activities to the crisis. In Bangladesh, Benin and Haiti, for example, Médecins du Monde is supporting the development of national pandemic plans with awareness-raising and prevention measures in community settings.

Raising the political profile of the challenges that pandemics bring will require a recognition of the political, security, poverty, gender, and developmental dimensions of public health emergencies. The inherent multidimensionality of the pandemic necessitates a coordinated multisectoral and multistakeholder response, including governments, UN agencies, and other non-state actors, such as civil society and the private sector, to link frontline infectious disease responders to a broader humanitarian and development community [21].

The example of Georgia demonstrates how a political commitment, effective public health response, and policy coherence across sectors determined the successful handling of the COVID-19 pandemic. At the time of writing, this small Caucasus country of 4 million people managed to keep the death rate relatively low at 28, out of more than 5,000 reported cases [22] (**Box 2**).

**Box 2: No silver bullets: Strong political leadership, multisectoral collaboration, compliance with IHR requirements, and public education were the contributing factors in Georgia’s successful response to COVID-19.**

To date, Georgia has performed well in its response to the COVID-19 pandemic, with a relatively low number of cases and mortality. Georgia’s success is likely due to a number of factors: The government started preparing early and has maintained a good level of public confidence in its activities; the response has been multisectoral; the public health community has led from the start; Georgia followed all IHR requirements from the very early stages of the epidemic; monitoring and surveillance of the infection, early laboratory detection and diagnostics, contact tracing, forecasting, daily reporting to the government and awareness-raising in the population through the mass media were performed at a high professional level.

The health system has been strengthening its human resources for health for a number of years, so the frontline workers were well-prepared for the emergency. In addition, Georgia mounted a multisectoral response involving not only the health sector but also the ministries of education, finance, foreign affairs and border security.

The leading Georgian public health institution – the National Center for Disease Control and Public Health – continually monitored the best practices of major world health institutions, such as WHO, Public Health England, the BMJ, the European Centre for Disease Prevention and Control (ECDC), the US CDC, the Chinese CDC, the Robert Koch Institute, and the Bunderswehr Institute of Microbiology, and incorporated best practices from these entities into the fight against COVID-19. The fact that the public health community in Georgia led the response to COVID-19 was critical to the country’s successful response to the outbreak.

In sum, a strong public health leadership from the beginning of the outbreak, policy coherence and multisectoral collaboration, implementation of IHR at the national level, international cooperation and community engagement through public education campaigns were important determining factors in slowing the spread and managing the infection throughout the country.

## 3. Focus on vulnerability for resilient communities

It is well known that socioeconomic and health inequities amplify health risks for poor and marginalized populations. By some estimates, one in three annual deaths globally, that is, over 17 million deaths, are avoidable if countries introduce measures to address health inequities and the underlying determinants [23].

The experience with pandemic influenza suggests that vulnerable populations serve as a familiar clinical index of the severity of a pandemic virus [24]. Indeed, the COVID-19 pandemic demonstrated that the crisis has exacerbated pre-existing chronic conditions and resulted in poor health outcomes in many countries. A recent meta-analysis revealed that hypertension, diabetes, chronic obstructive pulmonary disease (COPD), cardiovascular and cerebrovascular diseases – so-called non-communicable diseases – are major risk factors for patients with COVID-19 [25]. The higher prevalence of these conditions in older people, often combined with increased frailty, explains much of the higher mortality in this group.

In Italy, more than two-thirds of hospitalized patients had diabetes, cardiovascular diseases or cancer, or were former smokers [26]. In addition, people with COVID-19 have a higher likelihood of depression and lower health-related quality of life [27].

Other important vulnerable population groups at a higher risk are refugees and migrants, as these populations are overrepresented in camps and among the homeless. Living conditions for these vulnerable populations are most affected by income and health insecurity, unemployment, access to basic hygiene measures and health services, and restricted ability to self-isolate or quarantine. It is also worth noting that 80% of refugees live in low- or middle-income countries (LMIC), the countries that are expected to experience the fourth wave of COVID-19, after China, Europe, and the USA [28]. People living with disabilities is another group experiencing high vulnerability during public health emergencies.

Explicitly including vulnerable populations in pandemic preparedness plans will help reduce disproportionate negative outcomes for them and will also support a more equitable distribution of health across different groups, while seeking to maximize overall population health. This will also support building the resilience of communities as a whole so they will be better protected against the disruptions that future disease outbreaks may bring.

## 4. Sustained Public Health Communication and Health Literacy as a Primary Prevention Strategy

Risk communication and sustained public health information campaigns are a big part of emergency preparedness and effective pandemic response. However, the uptake of information is dependent on literacy rates, cultural sensitivities, pre-existing beliefs, and familiarity with basic science (e.g. the germ theory of disease) [29]. The COVID-19 pandemic has highlighted the fact that the lack of health literacy is “an underestimated public health problem globally [30].”

Health literacy, defined as “the degree to which individuals have the capacity to obtain, process and understand basic health information and services needed to make appropriate health decisions [31],” is a prerequisite for adopting healthy behaviors that can prevent both communicable and noncommunicable diseases. In Europe, nearly half the adult population is reporting having problems with health literacy and a lack of relevant competencies to take care of their health and that of their families [32].

Low health literacy is linked to poor hygiene practices at the individual, household, community, and institutional levels, which may lead to or exacerbate disease outbreaks. Handwashing with soap has been recognized as one of the most cost-effective health interventions to reduce the burden of disease. Yet, only 19% of the global population is estimated to wash their hands with soap after using sanitation facilities [33].

Efforts to improve population health literacy can bring tremendous benefits in terms of building populations’ resilience to health threats, promoting healthy lifestyle changes, and empowering individuals to make informed health decisions. Decision-makers have a responsibility to identify the health literacy needs of their populations and invest in sustained public information campaigns as part of pandemic preparedness and response. A highly effective public communications campaign has been critical to New Zealand’s success with its pandemic response to date (**Box 3**).

**Box 3: Building public trust and confidence: sustained clear communication was a key public health intervention in New Zealand’s successful pandemic response, especially in achieving high levels of cooperation with a countrywide ‘lockdown.’**

The New Zealand Ministry of Health started daily media briefings on 27 January, a month before the country’s first case of COVID-19 on 28 February. These briefings, led by senior public health officials, informed the population about emerging evidence and experience in other countries to ensure, *inter alia*, that the population knew what was happening and what might happen next.

The country had a comprehensive pandemic influenza plan. However, it quickly became apparent from the experience of other countries that a move from ‘stamping’ out the virus to ‘managing it’ would not only have a significant impact on the health and wellbeing of the population, it would also likely compromise the health care system and have a sizable economic and social effect. Health and other government officials worked closely with political leaders to build confidence and trust across the country in the core public health interventions (testing, contact tracing, and isolating cases and potential contacts) and in pursuing a strategy of elimination involving substantial border restrictions. A sustained public communications campaign with clear and simple messages helped people to understand the actions underway and provided practical advice on what people could do to look after their own and their family’s health. When the Government announced and implemented in March a strict nationwide ‘lockdown’, requiring most people to stay at home for at least four weeks, there was a high degree of public support (over 90 percent) and co-operation, despite the considerable impact on people’s lives and livelihoods. The simple message of “Stay home; Save lives; Be kind” was highly effective in unifying people behind the lockdown, which was successful in breaking the chain of COVID-19 transmission across the country.

## Conclusion

While the world is still waiting for a much-needed vaccine and treatment against COVID-19, now is the time to rethink and ‘reboot’ our approach to pandemic preparedness by strengthening public health systems and multisectoral coordination and their links to the IHR.

Countries vary in terms of geographic and population sizes, the level of economic development, epidemiological profiles, political systems, health systems maturity, and other contextual factors influencing their ability to plan and respond to epidemics. However, pandemic preparedness should be part of a broader public health strategy in any setting, and long-term investments will be required to strengthen core public health capacities even after the current pandemic abates and other priorities emerge. This will contribute to building resilient communities capable of enduring the unavoidable public health threats of tomorrow, including the shocks of pandemics.

There is no good substitute for prevention. Strong organized efforts by multiple stakeholders, including governments, civil society, international, public and private organizations, and individuals at all levels, will be key not only to improving pandemic preparedness but also to achieving sustainable strengthening of health systems for a healthier future and well-being for all.
